# Flexible and Polarization Independent Miniaturized Double-Band/Broadband Tunable Metamaterial Terahertz Filter

**DOI:** 10.3390/ma15228174

**Published:** 2022-11-17

**Authors:** Manikandan Esakkimuthu, Inbarani Jothinayagam, Karthigeyan Arumugam, Sheena Christabel Pravin, Mukesh Jewariya

**Affiliations:** 1 Centre for Innovation and Product Development, Vellore Institute of Technology, Chennai Campus, Chennai 600127, India; 2 School of Electronics Engineering, Vellore Institute of Technology, Chennai Campus, Chennai 600127, India; 3 Department of ECE, SSN College of Engineering, Chennai 603110, India; 4 CSIR—National Physical Laboratory, New Delhi 110012, India

**Keywords:** metamaterial, double band, tunable, conductivity, broadband

## Abstract

In this paper, the design of a double-band terahertz metamaterial filter with broadband characteristics using a single conducting layer is presented. The design uses a structured top metallic layer over a polyimide material. The proposed design has achieved broadband band-pass transmission characteristics at the resonances of 0.5 THz and 1.65 THz, respectively. The 3-dB bandwidths for these two resonances are 350 GHz and 700 GHz, respectively, which indicates that dual-band resonance with broadband transmission characteristics was obtained. The design has achieved the same transmission characteristics for two different orthogonal polarizations, which was confirmed using numerical simulation. The design was tested for a different angle of incidences and it was observed that this results in angle-independent transmission behavior. In addition, for obtaining tunable resonant behavior, the top conductor layer was replaced by graphene material and a silicon substrate was added below the polymer layer. By varying the Fermi level of graphene, modulation in amplitude and phase was observed in numerical simulation. The physical mechanism of double-band behavior was further confirmed by surface current distribution. The proposed design is simple to fabricate, compact, i.e., the size is λ_0_/8, and obtained dual-band/broadband operation.

## 1. Introduction

Wireless communication plays a significant role in societal development. It actually provides low latency and high-data-rate transmission for end-user applications [1]. Currently, researchers are looking for a spectrum to support high-data-rate communication. The next generation will be utilizing 5G and terahertz spectra for communication-related activities. For supporting high-speed data transmission, broadband communication, technologies and electronic components should be adopted [2,3]. Terahertz is the band of frequencies that lies in the far-infrared range between 0.1 THz and 10 THz. The THz spectrum is gaining attention in the field of research due to its variety of applications, including communication technology, medicine, industries and so forth [4]. The successful development of high power sources and detectors in the terahertz region leads to the further utilization of this spectrum [5,6]. Still, there is a specific need for active and passive components with specific characteristics, such as multi-band operation, high sensitivity, wideband operation, etc., for utilizing the spectrum further [7].

The filter is one such important passive component for providing a better signal-to-noise ratio in to the specified band of frequencies [8]. Band-pass filters are used widely for transmitting the desired frequencies with better selectivity while rejecting other signals [9,10]. These filters could be made by using metamaterial structures. Metamaterials are manmade sub-wavelength periodic structures that provide properties such as a negative index of refraction, epsilon-near-zero, perfect absorption, cloaking, increasing the gain of the antenna, etc., which are not provided by naturally available materials. It is mainly used in sensing, imaging, cloaking, etc. [11,12,13,14,15,16]. Many multi-band, wideband filters are reported in the literature for the THz region [17,18,19,20]. Generally, they involve the use of a multilayer structure, the modified ground plane for obtaining broadband characteristics. It is easier to obtain a fully broadband filter using a simple structure. However, achieving broadband characteristics in multi-band filters is difficult [21,22,23,24,25,26,27]. Additionally, tunable or reconfigurable passive components are in demand [28].

The reconfigurable or tunable properties were achieved by using different mechanisms, such as the properties of being mechanically tunable, electrically tunable, thermally tunable., etc. [29,30]. Graphene is a 2D material and the conducting properties could be changed by varying the applied Fermi energy. Graphene-based electrically tunable metamaterial-based phase modulator was reported [31,32].

In this paper, we present the design and analysis of a double-band terahertz band-pass filter with broadband characteristics using a simple metamaterial structure. The obtained results are compared with the state-of-the-art literature results. It is clear that the proposed structure achieved dual-band resonance with broadband behavior using a single layer patch and was designed with easily available materials. The effect of graphene on amplitude and phase modulation is also studied and discussed. The conductivity of the graphene was varied by varying the applied Fermi level and it indicates the use of the proposed structure in modulator applications. The proposed structure was designed and numerically validated using CST Microwave Studio software, 2022. The proposed structure has the advantages of low profile, i.e., compact (λ_0_/8, λ_0_ represents the wavelength corresponding to the first resonance), polarization independence, and angular stability. The transmission characteristics were obtained for both transverse electric (TE) and transverse magnetic (TM) modes. The TE and TM modes have no corresponding fields in the direction of propagation, and generally those fields are perpendicular to the direction of propagation.

## 2. Proposed Metamaterial Design

**Figure 1:**
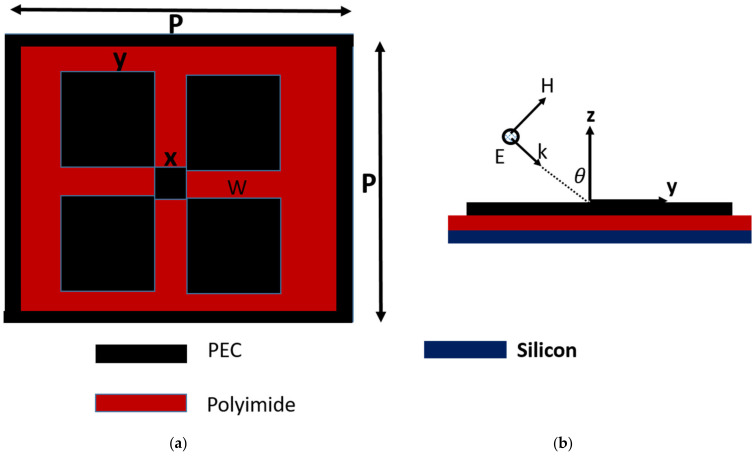
(**a**) Top view of the proposed THz metamaterial with geometrical parameters p = 75 µm, x = 7.5 µm, t = 5 µm, z = 9.375 µm, y = 16.875 µm, w = 26.25 µm. (**b**) Side view of the tunable metamaterial.

Figure 1a,b show the front and side view of the proposed dual-band terahertz metamaterial. The geometrical parameters are labeled within the front view of the structure. The structure consists of three layers, namely, a top metallic patch, a polyimide thin film, and a bottom silicon (Si) substrate. The thickness of the polyimide material and the Si substrate are 125 µm and 10 µm, respectively. The polyimide layer is assumed as a lossy one for numerical analysis. The polyimide dielectric properties, such as real permittivity (3.46) and the loss tangent (0.008) for the numerical analysis, were obtained from the literature, and the given data are measured at 1 THz. The tunable metamaterial design was achieved by replacing the metallic patch with a 2D graphene material. By adjusting the Fermi levels between the silicon and graphene patch, tunable transmission characteristics have been achieved [31,32,33,34]. In Figure 1b, the angle θ represents the incident angle, i.e., the angle between the incident wave and the normal to the proposed structure. 

## 3. Results and Discussions

The proposed structure was numerically evaluated by using a frequency domain method in CST Microwave Studio software. The evaluation uses unit cell periodic boundary conditions with incident waves normal to the frequency selective surface (FSS) structure. The characterization of the results includes transmission analysis, polarization behavior, angular stability analysis, modulation, and surface current distribution analysis.

### 3.1. Transmission Analysis

**Figure 2:**
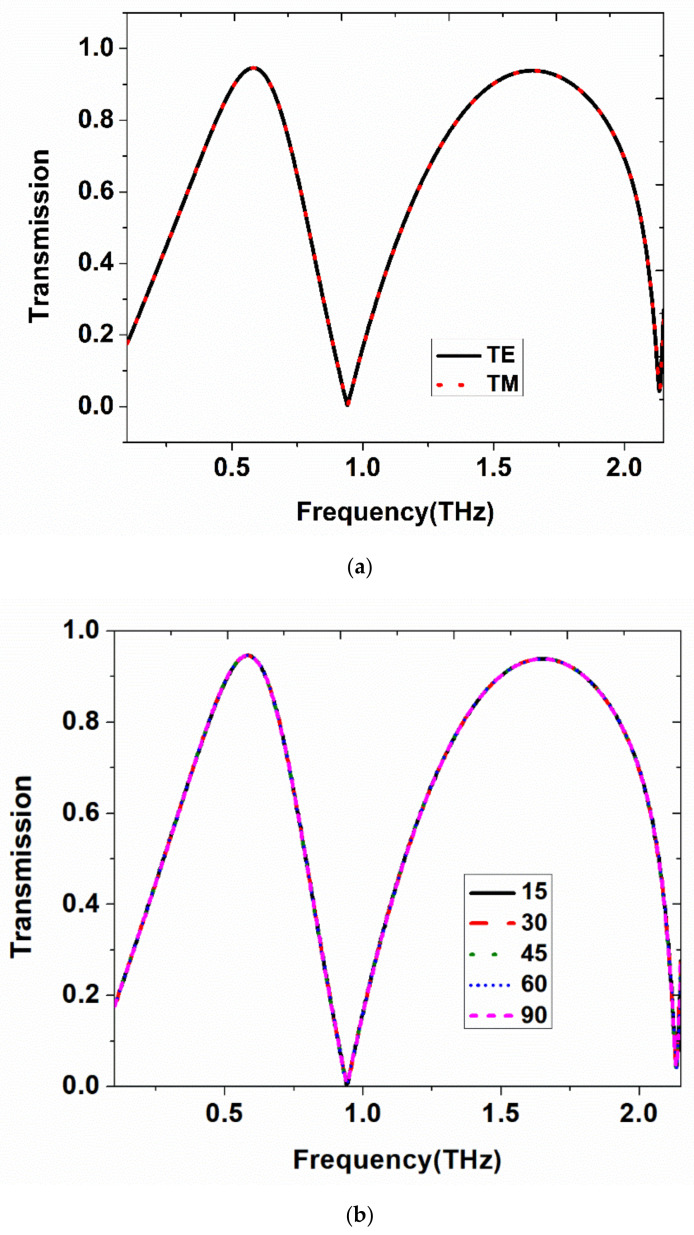

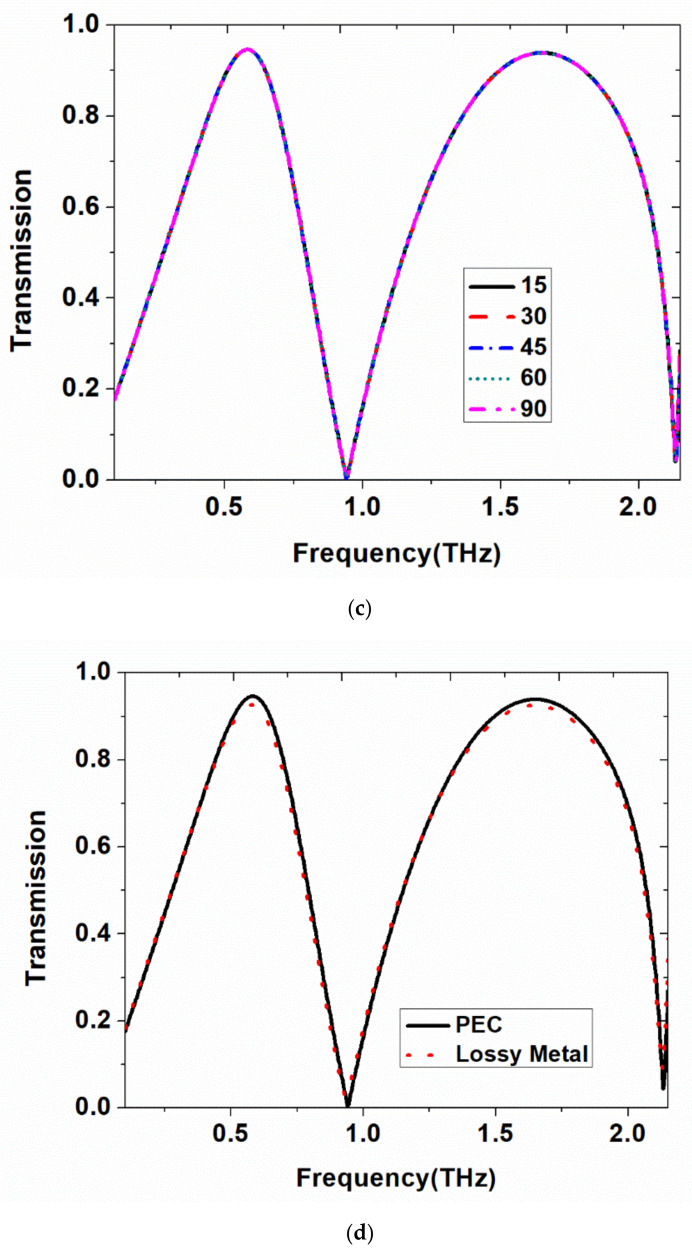
(**a**) Transmission characteristics of the proposed THz MM for TE and TM modes. (**b**) Transmission characteristics for different angles of incidence in TE mode. (**c**) Transmission characteristics for different angles of incidence in TM mode. (**d**) The proposed structure is evaluated for the lossy metal and compared with the PEC.

The proposed metamaterial structure resonates at two frequencies, namely 0.5 THz and 1.65 THz. The obtained transmission characteristic for two different orthogonal polarizations (TE and TM) is depicted in Figure 2a. It is confirmed that the designed structure has achieved polarization-independent transmission characteristics. The transmission of above 95% was achieved for the two resonances. The 3-dB bandwidth at the two resonances was 345 GHz and 700 GHz, which indicates the broadband transmission behavior.

For angular stability analysis, the design was simulated for a different angle of incidences in TE and TM modes, and their corresponding results are shown in Figure 2b,c, respectively. The structure is tested for different angles of incidence varying from 0° to 135°, and it should be noted that angle-independent transmission characteristics were achieved. Still, the structure shows better broadband band-pass characteristics for a wider angle of incidence. This weak angular dispersion was obtained by using a symmetrical resonator [35]. In terms of full width half maximum (FWHM), two resonances have achieved the bandwidth range of 510 GHz and 920 GHz, respectively. The transmission bandwidth of 130 GHz and 300 GHz showed more than 90% transmission at the two resonances, respectively. 

### 3.2. Physical Mechanism

For understanding the physical origin of the dual-band nature of the proposed structure, the simulated current distribution analysis is considered. The design has two resonators: an outer square and inside a butterfly-like structure. On simulating the outer resonator, the structure reveals a high pass filter characteristic. The inner butterfly wings structure without a center square show low pass filter behavior. By adding the inner wings with an outer square resonator, fully broadband band-pass transmission characteristics have been achieved. The comparison of the obtained results with the state-of-the-art literature works is shown in Table 1.

In addition, the metal is considered a lossy one at terahertz frequencies, and the simulation results were compared with the PEC-based analysis. This graph is depicted in Figure 2d. The parameters of lossy metal are set by the Drude model, ε_Au_ = 1 − ω_p_^2^/ω(ω + iω_t_), where plasma frequency ω_p_ = × 1.37 10^16^ rad/s, collision frequency ω_t_ = ×1.2 10^14^ rad/s [23]. It is observed that at lower terahertz frequencies, the metal behaves like a good conductor with a thickness greater than the skin-depth value at the desired frequency [36].

The minimum feature size in the proposed design is in the order of few micrometers and the design can be fabricated using ultra-short laser micromachining or standard wet etching facility. The conducting patch on the dielectric substrate can be deposited by using sputtering mechanism which is a standard and straightforward process of depositing thin film metals. This shows that the proposed design is simple and easy to fabricate and an array of them can be fabricated for sensor applications.

**Figure 3:**
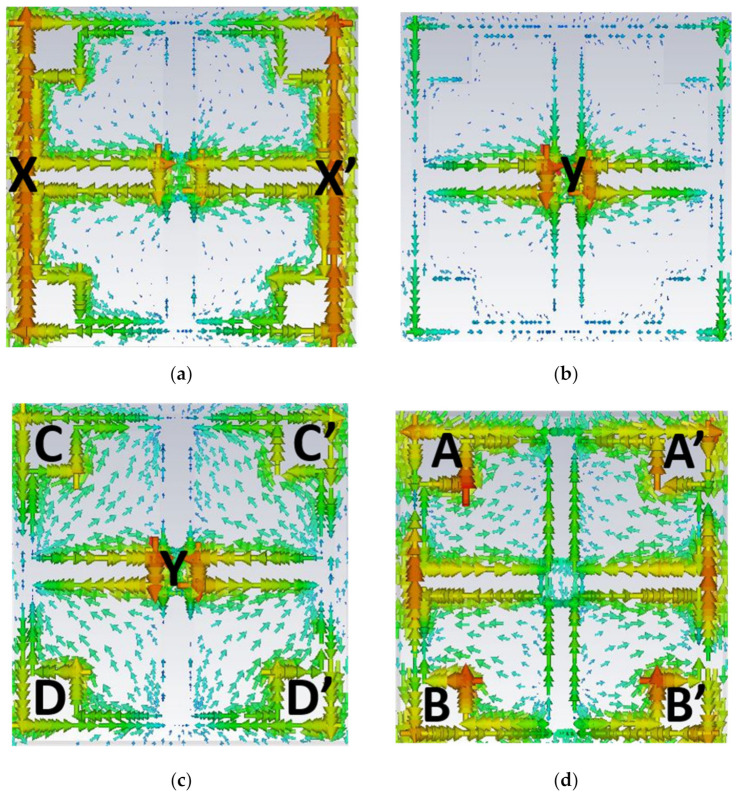
Simulated surface current density for the dual-band MM unit cell at the resonant frequencies (**a**) 0.5 THz, (**b**) 0.9 THz, (**c**) 1.65 THz and (**d**) 2 THz.

For obtaining dual-band characteristics, the center square resonator is attached to the entire structure, i.e., butterfly wings and outer square. The attached center square shows the notch band-stop characteristic. Thus, it has achieved the dual-band band-pass transmission characteristics with broadband behavior. Figure 3 shows the current distribution through the surface of the metamaterial.

The outer resonator contributes to the first resonance (0.5 THz) and the corresponding current distribution regions (X and X’) are marked and shown in Figure 3a. Figure 3b shows the obtained current distribution (region Y) at 0.9 THz which arises from the center square resonator. The butterfly wings along with the center square give rise to the second broadband resonance centered at 1.65 THz. The current distribution for 1.65 THz and 2 THz are shown in Figure 3c,d, respectively, and the corresponding regions are labeled. Through the current distribution analysis, the double-band/broadband filter characteristics are confirmed.

### 3.3. Conducting Characteristic of Graphene

Graphene is a 2D material with electrical conductivity that can be varied by altering the applied Fermi energy levels. The conductivity (σ) has two components, namely inter-band (σ_inter_) and intra-band (σ_intra)_ transitions, and it is calculated using the Kubo formula [32]. At terahertz and far-infrared frequency ranges, the inter-band transition is negligible and intra-band transition dominates (when ℏω < 2E_F_):

$$\sigma =\sigma_{intra}+\sigma_{inter}$$

where $\sigma_{intra}=\frac{ie^{2}k_{B}T}{\pi \hbar^{2}\left(\omega +i\tau^{-1}\right)}\left[\frac{E_{F}}{k_{B}T}+2ln\left(e^{\frac{-E_{F}}{k_{B}T}}+1\right)\right]$ and $\sigma_{inter}=\frac{e^{2}}{4\hbar }\left[\theta \left(\omega -2E_{F}/\hbar \right)-\frac{i}{2\pi }ln\frac{{\left(\omega +2E_{F}/\hbar \right)}^{2}}{{\left(\omega -2E_{F}/\hbar \right)}^{2}}\right]$.

If E_F_ > k_B_T and E_F_ > ℏω, the conductivity follows Drude-like form. Therefore, the conductivity can be deduced as follows:

σgra=e2EFπℏ2iω+iτ

where τ = uE_F_/(ev_F_^2^).

The conductivity of the graphene at THz frequencies is calculated based on the above-simplified formula and taken from the literature for the fixed Fermi velocity and carrier mobility [37]. These data were used for the numerical evaluation in this work. The concept of tunable conducting the nature of graphene is utilized for designing tunable metamaterial.

**Figure 4:**
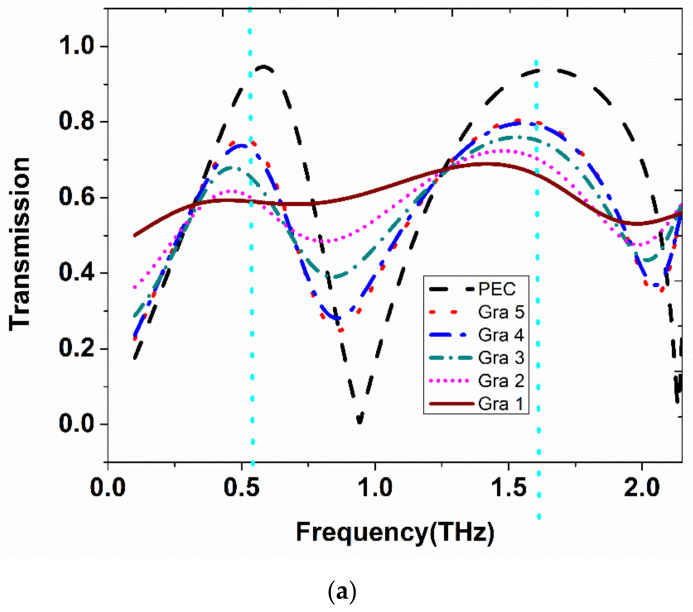

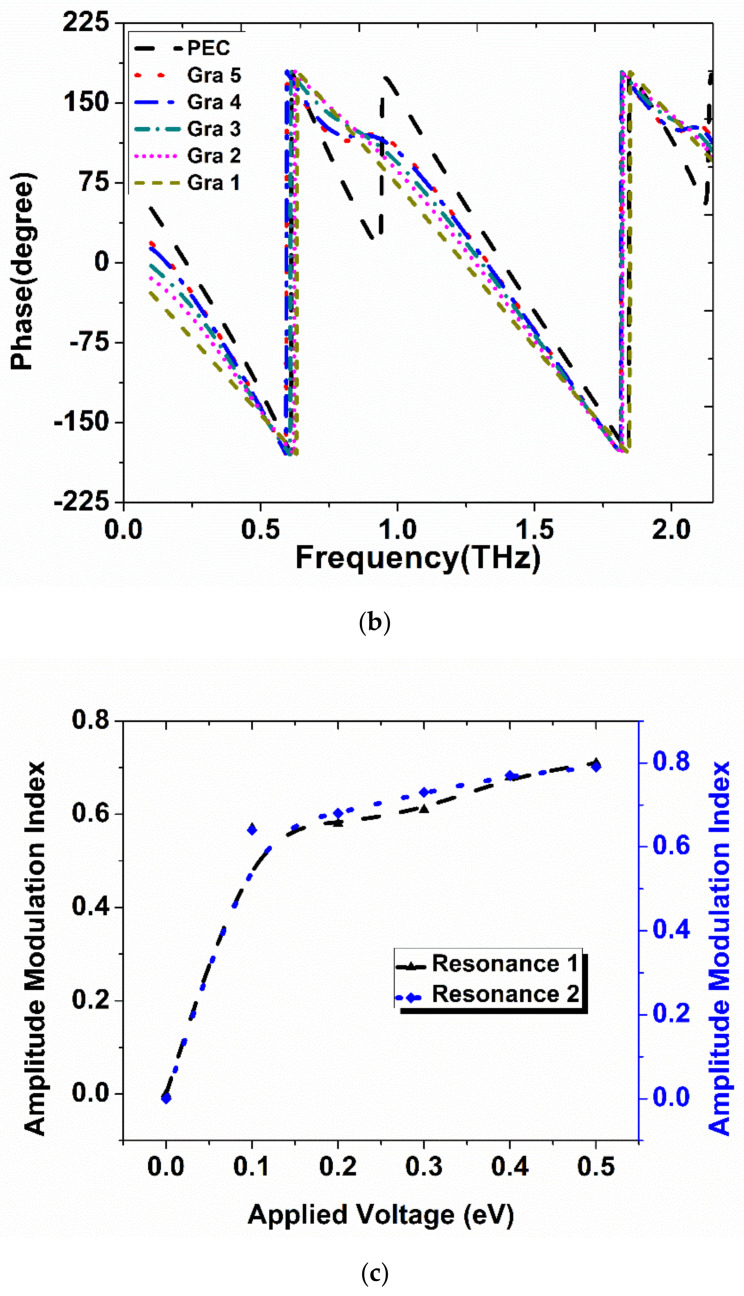
(**a**) Transmission spectra for graphene based MM for different Fermi levels Gra1 =0.1 eV, Gra2 = 0.2 eV, Gra3 = 0.3 eV, Gra4 = 0.4 eV, Gra5 = 0.5 eV, respectively (in addition to PEC). (**b**) The corresponding frequency versus phase characteristics. (**c**) Fermi level versus amplitude modulation index for the obtained two resonances.

Initially, the metamaterial was designed by considering the top metallic patch as a perfect electric conductor (PEC). Next, the patch was replaced with graphene material. The graphene conductivity values for different Fermi energy levels varied from 0.1 eV to 0.5 eV and were considered in numerical simulation. First, the designed structure was simulated for 0.1 eV and the corresponding transmission spectrum was obtained. Additionally, this continued until the 0.5 eV level. The obtained transmission spectra for these levels are obtained and plotted in Figure 4a. It should be noted that even for a smaller variation in energy level, the change in amplitude in the transmission spectra was significant. It confirms that the graphene-based proposed metamaterial will be useful for reconfigurable applications. The corresponding phase component for different Fermi levels is plotted in Figure 4b. This kind of metamaterial structure could be used for terahertz wave modulation.

The applied Fermi energy level versus amplitude modulation for the two resonances is depicted in Figure 4c. It is evident that the graphene-based metamaterial shows better modulation in amplitude as well as the in-phase component.

## 4. Conclusions

We have numerically evaluated a double-band terahertz metamaterial structure using a single layer structure. The designed metamaterial has obtained broadband operation in the desired resonances. The surface current distribution confirms the double-band resonance arises from the two resonators. From the transmission spectra, it is evident that the design has achieved polarization-independent and angle-resolved characteristics for two different orthogonal polarizations. The FWHMs for the two resonances are 510 GHz and 920 GHz, respectively, which further confirmed the broadband characteristics. The structure has achieved above 95% of transmission for both resonances. The tunable resonances have been achieved by replacing the metallic patch with graphene material. The graphene Fermi levels varied from 0.1 eV to 0.5 eV, and their corresponding spectra are discussed in this article. It showed better modulation for amplitude and as well as for phase characteristics. This kind of structure could be used in slow-light devices, filters, and so forth.

## Figures and Tables

**Table 1:** Comparison of the obtained results with the state-of-the-art literature.

Ref.	Dual-band	Broadband	Bandwidth	Reconfigurable	Polarization Insensitive	Complexity
20	Yes	No	26.4 GHz (f1), 23.5 GHz (f2)	Yes	Yes	No
21	Yes	Yes	257 GHz (f1), 8.3 GHz (f2)	No	Yes	No
22	Yes	Yes	500 GHz	No	Yes	No
23	No	Yes	~1 THz	Yes	Yes	No
24	No	Yes	2.9 THz	Yes	No	Yes, Multilayer
25	Yes	No	11 GHz (f1), 58 GHz (f2)	Yes	Yes	Yes, STO * structure
26	No	Yes	8 THz	No	-	Yes, Multilayer ERR *
This work	Yes	Yes	350 GHz (f1), 700 GHz (f2)	Yes	Yes	Single layer patch

* STO—strontium titanate, * ERR—electric ring resonator, f1-resonance 1, f2—resonance 2.

## Data Availability

Not Applicable.

## Acronyms

T	Temperature
E	Elementary charge
ℏ	Planck constant
$k_{B}$	Boltzmann constant
ω	Photon frequency
E_F_	Fermi energy in graphene
τ	Carrier relaxation time
θ(ω)	Expression for inter-band condition
v_F_	Fermi velocity
u	Carrier mobility

## References

[1] Liang L., Jin B., Wu J., Huang Y., Ye Z., Huang X., Zhou D., Wang G., Jia X., Lu H. (2013). A flexible wideband bandpass terahertz filter using multi-layer metamaterials. Appl. Phys. B.

[2] Elayan H., Amin O., Shubair R., Alouini M. Terahertz Communication: The Opportunities of Wireless Technology Beyond 5G. Proceedings of the International Conference on Advanced Communication Technologies and Networking (CommNet).

[3] Nadeem N., Parveen S., Ismail A. (2018). Terahertz Communications for 5G and Beyond. Antenna Fundamentals for Legacy Mobile Applications and Beyond.

[4] Tonouchi M. (2007). Cutting-edge terahertz technology. Nature Photon..

[5] Corsi C., Sizov F. (2014). Thz And Security Applications: Detectors, Sources And Associated Electronics For THz Applications.

[6] Jewariya M., Ragam S.R., Nagai M., Tanaka K., Abraham E., Yasui T. Generation of high power terahertz pulse using tilted wavefront technique and its prospectus in non linear terahertz spectroscopy and three-dimensional computed tomography. Proceedings of the 12th International Conference on Fiber Optics and Photonics.

[7] Kaur A., Myers J.C., Ghazali M.I.M., Byford J., Chahal P. Affordable Terahertz Components Using 3D Printing. Proceedings of the IEEE 65th Electronic Components and Technology Conference (ECTC).

[8] Ri-Hui X., Jiu-Sheng L. (2018). Double-Layer Frequency Selective Surface for Terahertz Bandpass Filter. J. Infrared Millim. Terahertz Waves.

[9] Wang D.S., Chen B.J., Chan C.H. (2016). High-selectivity bandpass frequency-selective surface in terahertz band. IEEE Trans. Terahertz Sci. Technol..

[10] Gallant A.J., Kaliteevski M.A., Brand S., Wood D., Petty M., Abram R.A., Chamberlain J.M. (2007). Terahertz frequency bandpass filters. J. Appl. Phys..

[11] O’Hara J.F., Singh R., Brener I., Smirnova E., Han J., Taylor A.J., Zhang W. (2008). Thin-film sensing with planar terahertz metamaterials: Sensitivity and limitations. Opt. Express.

[12] Shelby R.A., Smith D.R., Schultz S. (2001). Experimental verification of a negative index of refraction. Science.

[13] Craster R.V., Guenneau S. (2012). Acoustic Metamaterials: Negative Refraction, Imaging, Lensing and Cloaking.

[14] Cai W., Chettiar U.K., Kildishev A.V., Shalaev V.M. (2007). Optical cloaking with metamaterials. Nat. Photonics.

[15] Liang Y., Koshelev K., Zhang F., Lin H., Lin S., Wu J., Jia B., Kivshar Y. (2020). Bound States in the Continuum in Anisotropic Plasmonic Metasurfaces. Nano Lett..

[16] Pang K., Alam M., Zhou Y., Liu C., Reshef O., Manukyan K., Voegtle M., Pennathur A., Tseng C., Su X. (2021). Adiabatic Frequency Conversion Using a Time-Varying Epsilon-Near-Zero Metasurface. Nano Lett..

[17] Li Z., Ding Y. (2013). Terahertz broadband-stop filters. IEEE J. Sel. Top. Quant..

[18] Chiang Y.-J., Yang C.-S., Yang Y.-H., Pan C.-L., Yen T.-J. (2011). An ultrabroad terahertz bandpass filter based on multiple-resonance excitation of a composite metamaterial. Appl. Phys. Lett..

[19] Sun D., Qi L., Liu Z. (2020). Terahertz Broadband Filter and Electromagnetically Induced Transparency Structure with Complementary Metasurface. Results Phys..

[20] Ao T., Xu X., Gu Y., Chen Z., Jiang Y., Li X., Lian Y., Wang F., He Q., Zhou J. (2017). Terahertz band-pass filters based on fishnet metamaterials fabricated on free-standing SiNx membrane. Opt. Commun..

[21] Yan D., Meng M., Li J., Li X. (2020). Graphene-Assisted Narrow Bandwidth Dual-Band Tunable Terahertz Metamaterial Absorber. Front. Phys..

[22] Wang B.-X., He Y., Loua P., Xing W. (2020). Design of a dual-band terahertz metamaterial absorber using two identical square patches for sensing application. Nanoscale Adv..

[23] Zhang Y., Cen C., Liang C., Yi Z., Chen X., Li M., Zhou Z., Tang Y., Yi Y., Zhang G. (2019). Dual-band switchable terahertz metamaterial absorber based on metal nanostructure. Results Phys..

[24] Liu Y., Qian Y., Hu F., Jiang M., Zhang L. (2020). A dynamically adjustable broadband terahertz absorber based on a vanadium dioxide hybrid metamaterial. Results Phys..

[25] Wang T., Qu L., Qu L., Zhang Y., Zhang H., Cao M. (2020). Tunable broadband terahertz metamaterial absorber using multi-layer black phosphorus and vanadium dioxide. J. Phys. D Appl. Phys..

[26] Li W., Cheng Y. (2020). Dual-band tunable terahertz perfect metamaterial absorber based on strontium titanate (STO) resonator structure. Opt. Commun..

[27] Du C., Zhou D., Guo H.-H., Pang Y.-Q., Shi H.-Y., Liu W.-F., Su J.-Z., Singh C., Trukhanov S., Trukhanov A. (2020). An ultra-broadband terahertz metamaterial coherent absorber using multilayer electric ring resonator structures based on anti-reflection coating. Nanoscale.

[28] Mohammed H., Semih C., Mona J. (2017). Reconfigurable metamaterials for terahertz wave manipulation. Rep. Prog. Phys..

[29] Karl N., Reichel K., Chen H.-T., Taylor A.J., Brener I., Benz A., Reno J.L., Mendis R., Mittleman D.M. (2014). An electrically driven terahertz metamaterial diffractive modulator with more than 20 dB of dynamic range. Appl. Phys. Lett..

[30] Li J., Shah C.M., Withayachumnankul W., Ung B.S.Y., Mitchell A. (2013). Mechanically tunable terahertz metamaterials. Appl. Phys. Lett..

[31] Kim H., Charipar N., Breckenfeld E., Rosenberg A., Piqué A. (2015). Active terahertz metamaterials based on the phase transition of VO2 thin films. Thin Solid Films.

[32] Padilla W., Cich M., Azad A., Averitt R., Taylor A., Chen H.-T. (2009). A metamaterial solid-state terahertz phase modulator. Nat. Photonics.

[33] Sahin S., Nahar N.K., Sertel K. (2019). Dielectric Properties of Low-Loss Polymers for mmW and THz Applications. J. Infrared Millim. Terahertz Waves.

[34] Miao Z., Wu Q., Li X., He Q., Ding K., An Z., Zhang Y., Zhou L. (2015). Widely tunable terahertz phase modulation with gate-controlled graphene metasurfaces. Phys. Rev. X.

[35] Mahmud S., Islam S.S., Mat K., Chowdhury M.E.H., Rmili H., Islam M.T. (2020). Design and parametric analysis of a wide-angle polarization-insensitive metamaterial absorber with a star shape resonator for optical wavelength applications. Results Phys..

[36] Kirley M., Booske J. (2015). Terahertz Conductivity of Copper Surfaces. IEEE Trans. Terahertz Sci. Technol..

[37] Wang G., Zhang X., Zhang L., We X. (2019). Dynamically tunable plasmon-induced transparency based on radiative–radiative-coupling in a terahertz metal–graphene metamaterial. Crystals.

